# Supplementary material for: Long-term effect of middle ear disease on temporal processing and P300 in two different populations of children
Source: PLoS One. 2020 May 8;15(5):e0232839. doi: 10.1371/journal.pone.0232839 (PMC7209102; doi:10.1371/journal.pone.0232839)
Supplement: Attachment — Submitted filename: Rebuttal letter.docx [file pone.0232839.s002.docx]

PONE-D-19-32188

Long-term effect of middle ear disease on Temporal Processing and P300 in two different populations of children

Dear Reviewers,

Thank you for your valuable comments for which the Research Article titled " Long-term effect of middle ear disease on Temporal Processing and P300 in two different populations of children”, number PONE-D-19-32188, was submitted.

The authors accepted the majority of the suggestions made by the reviewers and the modifications made the article more interested and consistent for be published in this renowned journal.

We are available for further modification as needed.

Below are the comments for each reviewer regarding the suggested suggestions in bold and the number of the pages of each changes in the manuscript:

Review 1

This manuscript examines the long-lasting effects of otitis media in childhood on behavioral temporal processing and on a cortical correlate of attentional elements: P300 latencies and amplitudes. A major point of the study is to compare Australian and Brazilian children with a history of OME on these measures. They found effects of OME on P300 latencies, and on temporal processing, with the latter differing between the two populations. The basic study design is fine, but I have a number of concerns with the manuscript.

**We appreciate for the careful review of the article. We try to attend all the suggestions.**

1) The statistics are not appropriate for the study design. First, they are not described clearly: you mention that ear side and gender effects were included as interactions, and that group, gender and ear side were fixed variables. This implies that you used an ANOVA, but you didn’t describe this in the Results (no mention of main effects or interactions, either significant or not). Also, it is unclear what you mean by group: OME history, or location of subjects (Br or Aus)?
The appropriate analysis would be a repeated measures ANOVA, with fixed effects of study location, OME history, and gender, and with ear side as a repeated measure. If there is no effect of gender, you would then include this as a random factor in the ANOVA. You’d be expecting a main effect of OME history and study location, and possibly an interaction of the two. It is not appropriate to use t-tests in this study.

Importantly, it is not appropriate to double your N by analyzing ears independently and thus counting 2 ears per subject. This is particularly true because you are appropriately positing that OME causes central changes. The correct N is subject, not ear. Using ear as a repeated measure would solve this problem.
Throughout the manuscript, please report your F and df values, not just p values.
You describe that you used Pearson’s chi-square to test homogeneity of contingency tables, but you don’t report these results.

**The statistical was reviewed and ANOVA was used. (changes are in line 194).**

2) An important missing element that would lead to a better understanding of the data is the hearing loss experienced during OME. In an analysis of the human literature, Whitton & Polley (2011) showed that studies correlating early bouts of OME with later speech problems were equally likely to find or not find a correlation. In contrast, studies correlating the amount of hearing loss associated with those early bouts of OME with later speech problems almost always found a correlation. This is consistent with the broad literature and with your point that early auditory deprivation is driving the central auditory and perceptual changes, not simply the OME (lines 269-274). You should thus report the magnitude of hearing loss during OME bouts across your subjects in both groups.
**This information was included in the description of both otitis media group. (changes are in line 135)**

3) The Brazilian group had poorer behavioral performance than the Australian group, but your conclusion for the reasons behind this difference is not strongly supported by the data. This is partly because, as mentioned above, you don’t report OME-induced hearing loss (a proxy for the amount of auditory deprivation each subject experienced). You conclude that socio-economic status is likely a driver of worse Brazilian performance. This doesn’t quite make sense, because low socioeconomic status would be expected to result in insufficient treatment for OME, yet the Brazilian cohort all had tympanostomy tubes. This suggests they were receiving appropriate treatment. You also mention that the Australian cohort may not have had as serious OME, since none of them received tubes. This suggests that the hearing loss associated with the OME was likely more extreme for the Brazilian group, which is more likely the reason for the poorer behavioral performance in this group. You can’t exclude that the Australian children simply had less hearing loss associated with OME, allowing the development of better temporal processing abilities. Further, you can’t claim socioeconomic status as a driver for the study location difference if your groups do not have similar levels of hearing loss. Again as mentioned above, it would help to include information on the hearing levels during OME in both groups.

**A new study was done comparing only children who had three episodes of otitis media and underwent only clinical treatment. Children with ventilation tubes were excluded from this sample. (changes are in line 135)**

**Regarding socioeconomic status, a hypothesis suggested to explain the poorer performance on the Brazilian group is due to the delay in obtaining treatment in the public service, thus contribute to prolong the hearing deprivation in this population. (changes are in line 342)**

1) Introduction line 59-60: You mention that there are raised thresholds at high frequencies that outlast periods of OM. It is also important to mention (and cite) the degree of hearing loss typically associated with active bouts of OM, since this auditory deprivation is what is likely (and what you’re positing) to have affected the central auditory system.

**This information is in line 60.**

2) Introduction line 102-104, “The auditory deprivation, as a result of episodes of OM in childhood, compromises the normal development and maturation of the brainstem, mid-brain and cortical structures along the auditory pathway.”: Citation 14 does not support this statement - you’ve cited a behavioral assessment. You need to cite papers that show changes in central auditory structures. Either use human ERP studies, or animal studies, particularly something that shows changes at the level of cortex - e.g., Yu et al., 2005 Large-scale reorganization of the tonotopic map in mouse auditory midbrain revealed by MRI. PNAS 104:12193-12198; and Takahashi et al., 2006 Transcranial fluorescence imaging of auditory cortical plasticity regulated by acoustic environments in mice. Eur J Neurosci 23:1365-1376.

**The reference was changed. (line 106)**

3) Introduction line 104-105: “Degraded auditory signals result in dys-synchronized activity at the auditory cortex” – Are you referring to smaller amplitudes of P1 or N1 responses? Please provide a citation supporting this statement.

**We referred to all waves of LLEAP.** **The sentence was re-written. (line 107)**

4) Methods: It would be very helpful to include more information about your subjects. Hearing loss during OME is the most important. Also helpful would be the time elapsed since resolution of OME, duration of OME bouts, and number of OME episodes. Knowing individual ages during OME would be helpful, because the effects of auditory deprivation depend on developmental stage. I recommend putting individual subject information in a table. Also, does the mean age reported refer to age at time of testing?

**We also would like to include all this information about the time elapsed since resolution of OME, duration of OME bouts, and number of OME episodes, but we didn´t find these details on the medical report. So is one limitation in our study described on Limitations. (line 350)**

**The mean age referred at the time of assessment.**

5) Methods: Please provide more information about the Musiek GIN test. It would be useful to have a citation for the published test, since this test is standardized. Did the gaps have surrounding ramps? This is important, as the lack of a ramp creates a click, which is an additional cue. How were the no-gap trials distributed amongst the gap trials? How many trials at each gap duration? What were the specific gap durations used? Were they presented in random order, or was this a descending order test? Were gaps presented monotically? Based on the Results, it seems that the GIN was presented twice, once to each ear.

**All the information was included and the reference was already cited in the first manuscript (line 164).**

6) Methods: Please provide more information about the Musiek FPT test. Again, it would be useful to have a citation for the published test, since this test is standardized. How many trials were presented for each three-tone combination? Were they presented in randomized order?

**All the information was included and the reference was already cited in the first manuscript (line 171).**

7) Discussion, lines 267-268, “Similarly in animal studies, it was shown that 5-8 months of continuous bilateral ear plugging in ferrets resulted in a substantial elevation of temporal resolution ability.” Citation 36 is an abstract that doesn’t seem to have resulted in a peer-reviewed manuscript. Better citations for bilateral, reversible, early auditory deprivation in animals affecting temporal resolution could include: For gaps in noise, Green et al 2017, JNeurosci 37(32):7759 –7771. For amplitude modulation detection, Caras & Sanes 2015, JNeurosci 35(30):10831–10842.

**The reference was revised. (line 274)**

8) Discussion lines 293-295, “The association between OM and P300 may be explained by the fact that during the OM period the auditory cortex receives reduced auditory input from the brainstem which, as consequence, effects the auditory processing at the cortical level.” Please provide a citation for effects at the cortical level as a results of reduced auditory input. Animal studies could do, for example: Rosen et al 2012, PloS One, 7(7):e41514; and Yao & Sanes 2018, eLife, 7:e33891.

**The study suggested was included. (line 301)**

Typos/spelling/grammar:

1) Don’t capitalize Group on line 37
2) Don’t capitalize Otitis Media on line 39
3) Line 50: ‘although differences exist’ – this sentence is confusing, because it’s unclear what differences you’re referring to with this phrase.
4) Line 235: “significantly lower responses” is ambiguous for the GIN test, where a ‘lower’ response could be a shorter duration gap detection threshold. Perhaps use “significantly pooer performance”.
5) Lines 325 and 340: misspelled tympanostomy
6) Line 325: ‘failure’ should be ‘fails’
7) Line 341: misspelled ‘suggesting’

**All misspelling have been corrected.**

Review 2

**We appreciate for the careful review of the article. We try to attend all the suggestions.**

This manuscript is a long way from publishable in its current form. The main problems are as follows. The aim of the article is doubly unclear. Why have the particular tests of FPT, GIN and P300 been chosen? The introduction does not provide any compelling reason why these, out of all the tests commonly used in APD batteries, have been selected. They have the advantage of being non-language based, but was this the reason they were chosen?

**The non verbal tests were chosen to avoid language influence.**

Were there other tests carried out that were not reported because they had less interesting results?

**No**

The literature review needs to be more complete and clearer in its aim. Do the authors form the view that on the basis of previous literature, middle ear infections affect these test scores and electrophysiological response, and the primary interest is in whether it happens to a different degree in different countries? Or is there some doubt about the impact of middle ear infections on these measures, and the study is aiming to provide new knowledge on this issue?

**Our study would like to know how the episodes of otitis media affect the central auditory nervous system in different culture and how is this effect on the temporal processing and P300.**

Is the study aiming to establish the relative efficacy of two different medical treatments (antibiotics versus ventilation tubes) by investigating the later impact of each on auditory processing? If so, I have severe doubts about the validity of such a comparison given that the treatments were applied in populations that differ in other ways, the treatments were not randomised to participants, and the outcome variables are much less direct indicators of success than the degree of hearing loss that must mediate (.e cause) the outcomes measured in this experiment.We need a clearer aim.

**The purpose was re-written. (line 111)**

The populations chosen also need some justification. Apart from the obvious difference of being from two different countries, they differ in two ways. The Brazilian children presumably have lower socioeconomic advantage than the Australian children (which the authors have previously shown impacts on auditory processing test scores – Borge et al., 2013). They also have all had ventilation tube surgery, in contrast to the Australian children for which this is not the case. Is any difference found between the groups due to the difference in medical management, which in turn may have been due to a difference in severity or duration of infection, or is it due to a difference in the socio-economic advantage from which the populations are drawn? With this design and analysis method it is impossible to tell, so the results do not create worthwhile knowledge. In short, if this is a study of population differences, the populations should have had the same inclusion criteria, and if it is a study of treatment differences, the treatments should have been carried out on the same population (and preferably be randomly assigned).

**A new study was done comparing only children who had three episodes of otitis media and underwent only clinical treatment to avoid any questions about medical management. Children with ventilation tubes were excluded from this sample. (changes are in line 135)**

It is possible to partly address the preceding limitation with a more appropriate analysis method. The study includes control groups for each country, so on the assumption that these control groups also differ between countries in socioeconomic advantage, the interaction term in a two-way ANOVA with groups and countries as the two factors would provide a test of whether middle ear infections have different effects, relative to controls, in the two countries. Currently the paper rests on showing that the control groups do not differ significantly, but the otitis media groups do. This is not a valid statistical way to show that the effects of otitis media differ between the two populations, but the interaction term in an ANOVA is. Apart from the statistical analysis method being inappropriate, the results are just plain wrong in some places. Details are below under specific comments.

**The statistic was reviewed and ANOVA was used. (changes are in line 194).**

The other problem, more easily fixed, is that the written expression has to be improved in many places. Some sentences are not complete. The definite article “the” is missing in some places where it is needed and present in some places where it is not needed. One of the co-authors, or someone else with good written English ability will have to edit it after all revisions are made. There are so many places that need fixing, I have not commented on them.
Overall, I am uncertain whether the paper can be made publishable. The answer will depend on whether the authors can give the paper a clearer aim, which will be guided by a more comprehensive or perhaps focussed review of what is already known about the impact of otitis media on these two specific behavioural auditory processing tasks and on the P300 response.
**The english has been revised and all misspelling have been corrected.**

Specific comments
Line 44: It’s confusing to refer here to “a behavioural temporal patterning task”, when earlier in the abstract it is already called the FPT.

**We change for FPT.**
Line 48: “Lower responses” is ambiguous for the GIN, as lower thresholds represent better performance.

**This suggestion was accepted. (line 169)**
Line 50 and line 295: “effect” should be “affect”.

**This misspelling has been corrected.**

Line 78: A relevant additional reference would be Graydon, K., et al. (2017). "Consequences of Early Conductive Hearing Loss on Long-Term Binaural Processing." Ear Hear 38(5): 621-627.

**This reference was included. (line 80)**
Lines 81 to 91: This is a poorly written and not very informative paragraph. The first sentence is not a proper sentence. The definition of temporal processing in lines 84 to 85 does not add any meaning to the term. All acoustic stimuli are processed over time, so it is much better to focus on explaining the types of temporal processing that are intended, which the paper does elsewhere. The information in lines 85 to 91 is too vague to be useful. In line 90, it is not clear if it is past or current middle ear disease that is being discussed. Consider deleting the whole paragraph.

**The paragraph was re-written. (line 83)**
Line 98: Unclear meaning: The auditory cortex does not “investigate” anything.

**This information was removed.**

Line 102: Please supply a reference supporting the functions that the P300 is said to be associated with.

**The reference was included. (line 101)**

Line 105: Please supply a reference supporting the lack of synchronisation in the auditory cortex. Is it really known that it is a lack of synchronisation that is the cause of reduced amplitude responses, if that is what the observation actually is?

**The reference was included. (line 105)**

Lines 102 to 107: In these lines (which would be better as a separate paragraph), complete auditory deprivation (a “lack of auditory experience”) seems to be treated as having the same effects as the attenuation (that can be very mild) associated with otitis media.

**This sentence was re-written. (line 102)**
Lines 108 to 109: The sentence beginning “It can be” is not a proper sentence.

**This sentence was re-written. (line 107)**
Lines 108 to 112: This para implies, but does not actually state, that the purpose of the paper is to investigate the impact on auditory processing of different treatment for otitis media by comparing two populations for whom different treatments have been given.

**This paragraph was included to support the introduction.**

Lines 159 to 163: Were the GIN stimuli delivered to each ear separately?

**Yes. This information was included. (line 166)**

Line 171: The children had to hum and verbalise. Which response type was used as the basis of scoring? I infer from Table 1 “naming”, but not from Table 2 or 3, that it was verbalising.

**Just verbalising. This information was included on table 2 and 3.**

Line 174: LLEAP has become LAP, and this line should be after the heading it currently precedes. Easily spotted mistakes like this makes me wonder if the co-authors read the final manuscript.

**The suggestions were made. (line 178)**
Line 179: “2-channels” should be “two channels”.
Line 180: “monoaurally” should be “monaurally”.

**The corrections were made. (lines 183 and 184)**

Lines 191 to 195: The basis of the ANOVA must be group (OM versus control) by population (Brazil versus Australia). Ear can probably be added as a third repeated measures factor. The advice of a statistician should be sought as to how ear should be included, because of the very high correlations that are usually found between the two ears for both the GIN and FPT tests, though I think this does not prevent ear being added as a repeated measures factor. The t tests should not be used in isolation because of the confounding of socioeconomic advantage and treatment type that is currently present. A statistician could also advise how to include gender if that is needed. If including it in the analysis is a problem, it could be ignored if previous research indicates no gender effect for these tests.
Tables: the results should be arranged differently to better take advantage of the control groups, and to be consistent with the ANOVA I am suggesting. Each table should show the results for just one test (e.g. FPT), and that table should show all four scores: control and OM groups, by Brazil and Australian populations. Alternatively, show the same four scores (and their confidence intervals rather than box plots) in a figure.

**The statistic was reviewed and ANOVA was used. Also the tables were changed (changes are in line 194 and 209 to 241)**

Line 208: Typo in “mec”, plus put a space between numbers and their units, so “1.5 ms”.

**This correction was made.**
Table 2: The p value of 0.067 is not consistent with the means, SDs and N given. The correct answer is either 0.53 or 0.64 depending on whether one assumes equal true variance for the two groups.
Table 4: The p value of 0.060 for latency is not correct. It should be 0.36. Note that I have not checked all the statistical probabilities reported, just these two, since they were so obviously inconsistent with the data leading to them.
Line 224: Table 3 is actually Table 4.

**These corrections were made.**

Lines 256 to 257: This definition is too vague to be useful. Better to stick with defining the specific types of temporal processing investigated (gap detection and temporal order).Line 259: For which of the four types of temporal processing is this statement true? Be specific.
Line 266: Reverts back to temporal processing in general. Restrict the discussion to specific types of temporal processing, especially as the different types have almost nothing in common with each other.

**The sentence was re-written. (line 255)**

Line 287: Should be effect of “OM on P300”, not “OM and P300”. (This is just one example of inappropriate grammar; I have not commented on the rest.)

**This correction was made. (line 291)**

Lines 290 and 292: is line 290 talking about the presence of P300 responses and line 292 the differences in latencies? If so, it is a strange comparison.

**The sentence was referred to latency**.

Line 300: A lack of statistical significance in a difference does not imply there was no difference. That is why it is important to show the data, and important to test the two-way interaction term in an ANOVA.
Lines 305 to 306: The sentence is too general to be meaningful. If the FPT and GIN results differed across populations, then presumably the auditory cortex was affected differently by otitis media, even if this was not evident in the P300 responses.

**The sentence was re-written (line 316).**

Lines 310 to 315. This para contains non-informative generalities that do not help interpret the results of this experiment. Suggest deletion.

**The paragraph was excluded.**

Line 316: Give a reference to low-socio economic status increasing the severity of OM (even though I have little doubt it is correct).

**Béria, J.U., Raymann, B.C.W., Gigante, L.P., Figueiredo, A.C.L. Jotz, G. et al. Hearing impairment and socioeconomic factors: a population-based survey of an urban locality in southern Brazil. Rev Panam Salud Publica. Washington. June 2007; vol.21 no.6**
Line 321: “79 place” should be “79th place”.

**This misspelling has been corrected (line 330)**
Line 325: Typo in “tympanostomy” and “failure” should be “fails”.

**This sentence was excluded.**

Line 332: “Grommet” would be fine if used throughout, but don’t change terms from “tympanostomy tube” at this point in the paper.

**This sentence was excluded.**
Line 334: What benefits? Given the results in this paper, should it be “alleged benefits”. If the benefits of tubes are truly established, then what is the point of this study?

**This sentence was excluded.**
Line 336: What is meant by “clinical treatment”. Does it mean antibiotics?

**Yes. The sentence was re-written. (line 335)**

Line 341: Or might the lack of tubes in the Australian children arise from a difference in beliefs by doctors about the relative benefits of antibiotics versus tubes (which does exist even within Australia)?

**The group with tube placement surgery was excluded.**

Line 341: typo in “suggesting”.

**Correction was made.**

Line 342: Yes, do it. It might actually be necessary to state more about medical management preferences in the two countries for the results of this study to be interpretable.

Line 349: Given the significant difference between OM groups in the two countries found for FPT and GIN, but not for P300, why do the authors recommend that future studies measure P300. I could imagine the opposite conclusion being reached: that relative to behavioural measures, P300 appears to be insensitive to the deprivation effects of otitis media.

**Future studies was recommended once was observed statistical difference on P300 between children with a history of otitis media compared to children without middle ear infection.** **Thus, the OM affect the cortical responses**.

Line 375: Typo in “otitis”.

**Correction was made.**

Figs 1 and 2. These results should be combined to a single figure. That way the reader can compare results across the two countries, as well as between groups within each country.

**The figures were separated because the results were presented by topics: BrControl x BrOM and AusControl x Aus OM**
