# Supplementary material for: Long-term effect of middle ear disease on temporal processing and P300 in two different populations of children
Source: PLoS One. 2020 May 8;15(5):e0232839. doi: 10.1371/journal.pone.0232839 (PMC7209102; doi:10.1371/journal.pone.0232839)
Supplement: Attachment — Submitted filename: Response to Reviewers.docx [file pone.0232839.s003.docx]

PONE-D-19-32188

Long-term effect of middle ear disease on Temporal Processing and P300 in two different populations of children

Dear Editor,

Thank you for your valuable comments for which the Research Article titled " Long-term effect of middle ear disease on Temporal Processing and P300 in two different populations of children”, number PONE-D-19-32188, was submitted.

The authors accepted all the suggestions made by the editor and the modifications made the article more interested and consistent for be published in this renowned journal.

We are available for further modification as needed.

The manuscript was fully revised by an English native speaker and the changes were highlighted in yellow.

Below is the answer for each comment in bold and the number of the pages of each change in the manuscript:

Editor Comments:

1. Material and Methods L.124: Were the two cohorts, from Brazil and Australia, age-matched?

**Yes, the age matched. The mean age of each group was described in the lines 142, 145, 148 and 151.**

2. L.134: A definition of Recurrent Otitis Media must be clearly depicted. The text is not clear in separating infectious acute otitis media (AOM) and non-infectious otitis media with effusion (OME). AOM can cause inner disfunction through middle ear/inner ear interactions, via bacterial toxins. Could this occurrence influence the results?

**A definition of OME was described in the Introduction: line 58. When we described children with Recorrent Otitis Media we wanted to highlight the number of episodes, but all these children had OME. In order to provide a better understanding we replaced ROM for OME. Line 135.**

3. L158: Were the audiologic tests carried out in similar conditions and with equal audiometric devices. Calibrations could have influenced the outcomes?

**We were very concerned about the devices calibration in both countries. The tests were applied by the same type of audiometric devices and ear phones to avoid any possibility of influence in the results. This information was included in the lines 156, 157, 159 and 160.**

4. Sometimes the scores reported in this type of central processing exams are examiner-dependent. Could the authors clarify how they did to harmonize the results from both centers?

**In both centers the GIN test and FPT were applied by the same examiner to harmonize the results. This information was written in the lines 180 and 181.**

5. L.228/9 It is confusing: No significant differences in behavioural responses for P300 were found and this is followed by a p<0.05.

**We wrongly written “for” and “p<0.05” instead of “and” and “p>0.05”. The correct sentence was rewritten in the line 233.**

6. L237/8: the same as above (L.228/9) applies.

**The symbol “<” was changed to “>” in the Line 242.**

7. L.272: elevation means aggravation? (Seems a literal translation)

**This change was made. Line 271.**
8. L.275/8: How is “severe” OME defined. Is it worse to have intermittent OME than persistent OME in respect to auditory deprivation?

**The term “severe” means how many episodes of OME the children had. In the article cited the author suggested that the cohort study did not have enough episodes to cause auditory deprivation and reduce temporal abilities. The sentence was rewritten in the lines 274 and 275.**
9. L.294: found means finding?

**We wrongly written the word “found” instead of “finding”. The correct sentence was rewritten in the line 293.**
10. L.300: temetrically?

**The correct word is telemetrically. Is was rewritten in the line 299.**
11. L.317/8 correct gramar

**The gramar was corrected in the line 317.**
12. L.332 correct gramar

**The gramar was corrected in the line 331.**
13. L.337: queues mean waiting lists?

**Yes. We replace the word queues for waiting lists in the line 336.**
14. L. 342/2 correct gramar

**The gramar was corrected. Lines 341 to 346.**
15. Ref #7 Author name must be revised

**The Author name was corrected.**
16. Ref #19 is incomplete

**The reference 19 was corrected**.
17. Ref #38 the journal reference is correct?

**Yes, it is corrected. Now ir Ref #39**
18. Ref #43 seems an unpublished information

**This reference was removed.**
19. Ref #46 is the only one with et al. Was it borrowed from elsewhere?

**This article has more than 6 authors.** **So after the sixth author we wrote et al.**
20. Ref #47 the url is missing

**This reference has been updated and rewritten.**

Journal requirements:

**All journal requirements were made.**
